# Amantadine at the Extremes: Neuropsychiatric Complications of Use and Withdrawal

**DOI:** 10.1192/j.eurpsy.2026.11791

**Published:** 2026-07-31

**Authors:** S. Adas, B. R. Carr

**Affiliations:** University of Florida, Gainesville, United States

## Abstract

**Introduction:**

Amantadine is widely prescribed in Parkinson’s disease (PD) for dyskinesias and motor fluctuations due to its dopaminergic and NMDA-antagonist effect. Although effective, its psychiatric effects span a wide spectrum. At one extreme, use is linked to hallucinations, compulsive behaviors, and affective lability; on the other end, abrupt cessation may cause delirium, catatonia, or neuroleptic malignant reactions. These contrasting presentations can obscure diagnostic clarity, as symptoms may be misattributed to PD progression, comorbid psychiatric illness, or neuromodulation effects.

**Objectives:**

To illustrate the psychiatric complications of amantadine use and withdrawal, identify risk factors for the presentations, and emphasize their relevance for psychiatric assessment and management in PD.

**Methods:**

A literature synthesis (PubMed, Google Scholar) was performed on amantadine’s neuropsychiatric outcomes, emphasizing hallucinations, psychosis, compulsive behaviors, delirium, and withdrawal syndromes. These outcomes were observed in the Parkinson’s disease population to highlight clinical presentation, risk factors and diagnostic challenges.

**Results:**

Amantadine use is associated with hallucinations (visual, auditory), paranoid ideation, compulsive behaviors (punding, shopping), and mood lability. These presentations typically were seen in the setting of long-term dopaminergic or psychotropic polypharmacy. Amantadine withdrawal due to abrupt cessation can cause cataonia, delirium and malignant-like syndromes, but symptoms often are reversible upon drug reintroduction. Risk factors include a high cumulative dose, prolonged use, preexisting psychiatric or cognitive impairment, and concurrent use of antidepressants or antipsychotics.

**Conclusions:**

Amantadine is an example of how pharmacologic intervention of dopaminergic-glutamergic networks can affect psychiatric functioning in both directions, whether through excess activity during treatment or lack off upon withdrawal. For psychiatrists, keeping this “extreme profile” in mind can help guide management since symptom presentations that appear to be Parkinson’s disease progression may be medication-related. Recognizing amantadine’s potential effects can prevent misattribution, guide timely management, and allow for effective interdisciplinary management in PD.

**Disclosure of Interest:**

None Declared

